# Walking to a pathway for cardiovascular effects of air pollution

**DOI:** 10.1016/S0140-6736(17)33078-7

**Published:** 2017-12-05

**Authors:** George D Thurston, Jonathan D Newman

**Affiliations:** New York University School of Medicine, Department of Environmental Medicine, Tuxedo, NY 10987-5007, USA (GDT); and New York University School of Medicine, Department of Medicine, Division of Cardiology, New York, NY, USA (JDN)

There is a well documented association between human exposure to fine particulate matter air pollution (PM_2.5_) and an increased risk of cardiovascular disease and death.^1,2^ Indeed, the Global Burden of Disease (GBD) study^3^ recently estimated that exposure to PM_2·5_ contributed to 4·2 million deaths in 2015, representing the fifth-ranked risk factor for global deaths; of these, mortality from cardiovascular disease (CVD; ie, ischaemic heart disease and cerebrovascular disease) accounted for most deaths attributed to ambient PM_2·5_ air pollution. However, despite these strong epidemiological associations and the documented widespread adverse health effects, the exact biological mechanisms and the types of particles that are most responsible for the PM_2.5_-CVD associations are not well defined.

In *The Lancet*, Rudy Sinharay and colleagues^4^ use a simple but elegant randomised crossover design to gain insight into the type of pollution that can lead to the air pollution-CVD associations that have been reported in population-based epidemiological studies, as well as to identify specific cardiovascular changes consistent with the causality of those associations. The researchers studied the effects of traffic pollution exposure in adult participants aged 60 years and older during a 2 h walk along a busy commercial street in London, England (Oxford Street) compared with a similar walk in a nearby London park (Hyde Park), which has much lower air pollution. 40 healthy volunteers, 40 participants with chronic obstructive pulmonary disease, and 39 participants with ischaemic heart disease took part. In all 119 participants, irrespective of disease status, walking in Hyde Park led to an increase in lung function and a decrease in arterial stiffness, measured as pulse wave velocity and augmentation index, following the walk. By contrast, these beneficial responses were significantly diminished after walking along the more polluted Oxford Street. Specifically, among healthy volunteers the investigators reported a roughly 5% (95% CI −10·40 to −0·27) decrease in pulse wave velocity from 2 to 26 h after the Hyde Park walk, an exercise benefit that was not only negated but even reversed 26 h after the Oxford Street walk (7% increase in pulse wave velocity, 95% CI 2·16 to 12·20). Thus, the multifactorial benefits of low-to-moderate intensity physical activity, such as walking, for the primary and secondary prevention of CVD^5^ were offset by the presence of air pollution. Reductions in measures of arterial stiffness have been recorded with the use of guideline-directed medical therapy;^6^ however, until this study, evidence has been scarce on the adverse effects of air pollution exposure on vascular function during physical activity.^7^

Important to the interpretation of this study is the finding that air pollution causes phospholipid oxidation^8^ and oxidative stress (eg, by transition metals in fossil fuel combustion particles).^9^ These pathways accelerate atherogenesis and increase arterial stiffness, itself a strong predictor of cardiovascular events and all-cause mortality.^10^ However, one limitation of such panel studies is their size; as such, generalisability can be an issue. In view of this limitation, more and larger practical real-world exposure studies like the one done by Sinharay and colleagues^4^ that also assess novel in-vivo biomarkers of oxidative stress and phospholipid oxidation might further clarify the mechanistic pathways and clinical implications of air pollution exposure, and broaden their known applicability. Furthermore, additional evidence on the temporal relationships and longer-term cumulative effects of chronic air pollution on arterial stiffness is also needed. Overall, however, data from Sinharay and colleagues provide significant new evidence of an important biological pathway between subclinical CVD and the systemic effects of air pollution exposure.

The design used by Sinharay and colleagues is especially important in providing patient-level data for both parameters of air pollution exposure and measures of vascular function during a controlled period of physical activity. Significant increases were noted in arterial pulse wave velocity and augmentation index among the healthy volunteers that were associated with greater exposures to ultrafine particles and black carbon soot (markers of diesel vehicle emissions in this setting) but not with overall PM_2.5_ mass.^4^ These new results are consistent with our past investigation in urban children with asthma, which found that a worsening of respiratory symptoms was significantly associated with personal exposures to black carbon soot, but not with PM_2.5_ mass.^11^ The fact that the associations were more significant for the black carbon soot and ultrafine particle components of the PM_2.5_ also concurs with past indications that, of the PM_2.5_ in the ambient air, fossil fuel combustion particles are especially important to the associations found with CVD mortality and morbidity.^12^

The changes in arterial stiffness reported in the study by Sinharay and colleagues are biologically consistent with the air pollution and CVD health associations found in the population-based studies of hospital admissions and mortality, further strengthening the consensus that the association between particulate matter and CVD is causal. Although more studies are needed on the respective health effects of all the individual constituents and sources of PM_2.5_, the results of this and other recent urban studies already indicate that policy makers and health professionals should make the reduction in public exposures to diesel particulate matter a high priority in PM_2.5_ air pollution control and patient avoidance strategies.
